# “I am willing to do both well”: Chinese academic mothers facing tension in family and career

**DOI:** 10.3389/fpsyg.2022.973110

**Published:** 2022-11-03

**Authors:** Li Bao, Guanghua Wang

**Affiliations:** ^1^School of Sociology and Population Studies, Nanjing University of Posts and Telecommunications, Nanjing, China; ^2^Faculty of Education and Social Work, University of Auckland, Auckland, New Zealand; ^3^International Programs and Services, Shanghai Normal University Tianhua College, Shanghai, China; ^4^School of Education, University of Hertfordshire, Hatfield, United Kingdom

**Keywords:** Chinese academic mothers, career advancement, heterosexual matrix, gender binary, normativity, women academics

## Abstract

Academic mothers perform intersected roles. They carry out their profession in workplaces, while they take the “second shift” of motherhood back to their families. The contested expectations in family and career built by the heterosexual matrix cause tension to academic mothers. We qualitatively investigate the interview data of six Chinese women academics on how they perform to negotiate their motherhood and academic work in the context of Chinese higher education, driven by the Butlerian theoretical concept of the heterosexual matrix. The findings suggest that Chinese academic mothers play a zero-sum game between being mothers and being academics, deriving from their ontological responsibilities of motherhood. We conclude that in the masculine academia, these women academics help maintain the heterosexual matrix by satisfying the gender normativity when they negotiate their performances in their family and career; meanwhile, most have developed some strategies to achieve their career advancement.

## Introduction

Like other women academics worldwide, many women academics in China devote themselves to academic careers in universities and simultaneously hold the identity of being mothers back to families. Society expects mothers to be the main caregivers (Armenti, 2004; Manathunga et al., 2020), especially in the Chinese child-centered family culture (Liu, 2019). Childrearing, as one of the major obligations in marriage, consistently gives rise to tension between rational allocation in family responsibilities and individuals' successful career development (Cho and Ryu, 2016; Amsler and Motta, 2019). Meanwhile, as a quest, the central government of China aims to build stronger research-intensive universities (Rhoads et al., 2017), and hence, it urges academics to be productive, regardless of their life stages. By this means, careless institutional culture has been constructed in contemporary neoliberal academia, and therefore, it enhances constant work–life conflict, which navigates career paths of women academics.

Academic life in this careless culture emphasizes competition—individuals strive for rewards in short supply (Acker, 2010). Amsler and Motta (2019, p. 91–92) describe how the careless culture influences academics and what neoliberalism expects from academics:

*The ideal neoliberal subject is infinitely flexible, always on call, de-gendered, de-raced, declassed and careless of themselves and others. The onto-epistemological violence enacted against other ways of being is immense; attempts to erase all practices, imaginings and embodiments of becoming academic differently*.

The prior research on neoliberalization in universities often demonstrates the loss of autonomy among academics (Amsler and Motta, 2019). This careless context neoliberalism in higher education contradicts the caring work culturally associated with women (Lynch, 2010). Neoliberalism expects academics to be deindividualized and fully involved in academic work in a collective way, which blurs the academic and non-academic dimensions of life (Amsler and Motta, 2019) and leads academics to combine academic work with other commitments.

Researchers pay attention to the major conflict in relation to the tension among women academics: work–life balance. Many researchers explain that this dilemma is mainly attributed to the disproportionate family responsibilities allocation, and it leads to the underrepresentation of senior women academics because they may have less time for research (Aiston and Jung, 2015; Diksha, 2015; Dickson, 2018). Women academics' career and biological clocks are ticking simultaneously (Dickson, 2018) to urge them to become mothers in the early career stage. The allocation of time in a family is constrained by gender norms, and women are more likely to be the ones who make concessions (Diksha, 2015). Being the main caregivers in families, women academics, who have flexible working time, usually face little division between work and family affairs (Hagqvist et al., 2015). Although some academic mothers may gain simultaneous satisfaction with motherhood and academic careers in privileged working contexts (Huopalainen and Satama, 2019), many academic mothers tend to show negative feelings that family chores, childrearing, and academic workload are intersected so that they have to work anytime and anywhere, and they never get off (Dickson, 2018). Therefore, to tackle this problem, these women academics who are successful need to have strong family support in the first place (Cho and Ryu, 2016). For example, women professors who manage to climb up the academic ladder usually have supportive family members and domestic helps to promise them enough time (Ruan, 2021). The extent of tension between motherhood and career commitment depends on the availability of live-in home help with childcare (Dickson, 2018). However, in the COVID-19 era, the absence of external help and working from home during the lockdown highlight the tension in gender roles in childcare (Rania et al., 2022) and thus raise new and specific challenges to the work–life balance (Rosa, 2022).

Although women academics' intersection of roles in family and career has been broadly discussed for decades in international academia (Acker and Armenti, 2004; Aiston and Jung, 2015; Cho and Ryu, 2016; Dickson, 2018; Amsler and Motta, 2019; Chen and Hsieh, 2019; Aiston and Fo, 2021), how Chinese women academics are constructed and shaped by their responsibilities of being mothers is understudied. In the Chinese social context, women academics usually have lower career expectations and more work–life conflicts, which may lead to their low self-efficacy in career development (Cho and Ryu, 2016). They are somehow influenced by the Confucian culture of the subordinate status of women as incomplete and dependent beings to support the development of husbands and children (Rosenlee, 2006). Chinese academia shows expectations toward academics in the context of elite university construction as a national policy. In recent years, in the context of neoliberalism, some Chinese leading universities have implemented policies with “publish or perish” as a new trend, expecting high productivity from academics (Ren and Liu, 2021). Moreover, many Chinese women academics have strong agency for career progression and struggle to be recognized in academia (Bao and Tian, 2022). Their conflicting roles as women and academics require them to respond to different requirements (Suarez-Ortega and Risquez, 2014). Therefore, being academic mothers in China, especially in leading universities, is challenging.

In this study, we are interested in how Chinese academic mothers react to the tension when they face the intersected roles of academics and mothers. The potential of this work lies in two aspects: for one, this in-depth exploration of Chinese academic mothers may add to the quite limited qualitative studies on women in the Chinese higher education context; for another, the analysis of Chinese academic mothers' performances may enrich the understanding of gender practices in the neoliberal global academy. We aim to explore two research questions through the critical analysis of qualitative data collected by semi-structured interviews: (1) How do Chinese academic mothers perform in the contested normativity of being women and being academics? (2) How do Chinese academic mothers seek their career advancement under this tension?

## Theoretical framework: Heterosexual matrix

Female and male, woman and man, these discursively constructed concepts are descriptions as well as restrictions. The “either–or” alternatives announce the hegemony of heterosexuality. Assuming people are sexually divided, it is also impossible to identify masculine or feminine as monolithic and monologic “that traverses the array of cultural and historical contexts in which sexual difference takes place” (Butler, 2006, p. 18).

In the matrix of heterosexuality, gender is signified. For gender, heterosexuality is a prediscursive, ontological segregation. The segregation appears in two layers: first, it segregates heterosexual and non-heterosexual groups; second, it segregates men and women on the presumption of binary genders. As normativity, heterosexuality sets the traditional gender roles of, for instance, wife and mother, through the control and exploitation of women (Richardson, 2000, p. 20). Butler (2006, p. 26) argues that “the binary regulation of sexuality suppresses the subversive multiplicity of a sexuality” that forms heterosexual hegemonies. For society, this epistemological gender binary collectively categorizes individuals according to a prediscursively constituted ontological sexual frame.

The heterosexual matrix speaks for dualism. For Butler, “gender can denote *a unity* of experience, of sex, gender, and desire” (Butler, 2006, p. 30). This unity is built on the continuity of sex and gender and the heterosexual desire (Butler, 2006). These presumptions unravel “a stable and oppositional heterosexuality” (Butler, 2006, p. 31), which can only be “truly known and expressed in a differentiating desire for an oppositional gender, that is, in a form of oppositional heterosexuality” (Butler, 2006, p. 31). This division of binary gender is based on ontological sex, and gender develops from this sex origin in the heterosexual frame. Therefore, gender is more likely to be the representation of the intersected relations among sex, gender, and desire, and it results in forming these oppositional roles between women and men. Butler (2006) claims that heterosexuality is the cultural desire buried in social discourse. Its “either–or” binary working system defines its exclusion of the things that do not belong to either of both options with a clear boundary in between. The heterosexual matrix provides separated frames for women and men.

For academic mothers, they are constrained by the heterosexual matrix to be the main caregivers to satisfy the social expectation of women. In this way, they are likely to maintain “a stable and oppositional heterosexuality” (Butler, 2006, p. 31), and their gendered responsibilities are defined collectively, regardless of their desire for career advancement in neoliberal institutional culture. In this study, we seek to explore the performances of these Chinese academic mothers in the male-dominated social and institutional discourses using the Butlerian theoretical concept of the heterosexual matrix.

## Methodology

In this study, we employed critical discourse analysis of interview data collected through narrative inquiry. For data collection, we conducted feminist semi-structured online interviews with 20 Chinese women academics working in leading universities (ranked top 30 domestic universities) in mid-2020 in a larger research program granted by the University of Auckland Human Participants Ethics Committee (Reference protocol number: 024731) on the topic of Chinese women academics' career development, from which the data for this study were extracted. The interviews were conducted according to a carefully designed schedule and strictly followed the guidelines of the granting body. For example, participants were well informed of the relevant information *via* the formal consent form before the interviews. The first author was the main interviewer. The participants were asked open-ended questions, for instance, “How have you dealt with the obstacles in your career to date?” and “Have you felt being a woman ever positively or negatively affected your academic career development and why?” The participants were encouraged to talk about their lived experiences, especially in the context of Chinese universities. They were then probed with more follow-up impromptu questions based on their narratives and left with ample space to express feelings. We selected six of the participants (see Table 1) who elaborated on the tension of motherhood and academic career. Every one of the participants was assigned a pseudonym for ethical reasons. One of these women academics, Xixi, was single without children, but she provided a closely relevant narrative from her understanding. The other five women academics were mothers with one or two children. Given that most online interviews were conducted when these women academics were at home, some situation in their childcare was also included in the data analysis with the consent of the participants (in Han and Naya's cases). All the interviews were digitally recorded, transcribed, and selectively translated from Chinese to English for data analysis.

**Table 1:** Biographical information of the participants.

**Pseudonym**	**Working years**	**Academic rank**	**Children's age**
Han	4	Lecturer	1
Kadi	10	Associate professor	4
Ming	15	Associate professor	10
Naya	10	Associate professor	3;8
Xixi	2	Lecturer	/
Yang	8	Associate professor	6

We utilized the narrative inquiry method in this study to explore Chinese women academics' perceptions, values, and stances shaped by their experiences and stories, with one of the main purposes to describe their career development. As a primary way of presenting detailed information, this inquiry “called for the collection and organization of rich, descriptive stories” of women participants and provided “a strategy to interpret their stories in detail” (Sandekian et al., 2015, p. 364). In the narratives, the relationship the women have built on with culture, economy, and politics are reflected (Thorne and Varcoe, 1998). The “collusive or resistant strategies that narrators develop” (Chase, 2011, p. 430) to the constraints produced by gender inequalities are analyzed. On the other hand, narrative inquiry can be used as “a means of not being silent (or silenced) about difficult-to-articulate things (love, hope, disillusionment, death, and loss) in the current discursive environment” (Kelly, 2015, p. 1). In this study, the methodology of narrative inquiry enables the women academics to articulate their perspectives and interpretations from their contextualized agency (Pitre, 2011) to reveal their interactions with institutions and society. For the participants, the demonstration of their narratives originated from their competing desire of being good mothers and successful academics (Raddon, 2002). We had an open and tolerant attitude to interpreting their identity construction in the heterosexual matrix, as a temporal frame (Bochner, 2001) being articulated by the participants.

In this study, we employed critical discourse analysis to explain the data. We, as feminist critical researchers, aim to expose critique oppression, domination, and power inequities, which limit women's participation in society, politics, and economy (Kushner and Morrow, 2003). We intend to give voice to the silent group, reveal the invisibility, and “identify the interaction between social actors and their multifaceted world” (Pitre, 2011, p. 54). We interpreted the data in three steps: first, we performed preliminary qualitative data analysis and thematically sorted the transcripts of six participants and then categorized three overarching themes to answer the research question. Second, we further analyzed the discourse from a social ideological perspective of the Chinese context. Third, to investigate academic mothers' tension in a heterosexual matrix, we used a critical approach to the Butlerian theoretical framework in the data analysis and discussion, kept interactive with the theory (Hage, 2016), and examined this theory in a Chinese higher education context.

## Findings: Tension in the heterosexual matrix

The interviewed Chinese academic mothers reported their experiences of playing a zero-sum game between motherhood and academic career for their ontologically gendered responsibilities constructed by the hegemonic heterosexual matrix. If they emphasize career development, they need to obtain outward help. However, this support often comes with the guilt of not taking gendered responsibilities. We elaborate on the data analysis in the following sections.

### A zero-sum game for academic mothers

Participants agreed that being mothers and academics, as contested goals, were more likely to be a zero-sum game. Naya is an academic mother with two children. When we conducted an interview appointment with her, she replied, “*I am interested in this research, but I am afraid I can't start the interview until my children fall asleep at about 9, normally. Can I contact you then? I am not sure about the exact time*.” As the main caretaker of two children, Naya had to do her academic work after childcare. In the interview, she concluded family and career in retrospect:

*It is about family affairs. I think, in the comparatively long time, five to ten years, if you could hang on there, the torture of the family affairs would be time-limited…We, women academics, are attacked by the combo boxing of work, family and children. There is no other generation, I think maybe there is no other generation of academics live a harder life than us. (Naya)*

In the marketized institutional normativity, Chinese women academics are confronted with fierce competition to survive the neoliberal academia, along with the strategy of higher education development in China. At the same time, “traditional Asian culture places a premium on women as dutiful wives, mothers, and homemakers,” and it largely affects the behaviors of women academics (Aiston and Fo, 2021). This social context explains the metaphoric “*combo boxing*” described by Naya. In careless neoliberal academy, these Chinese academic mothers have to negotiate their multiple identities to fulfill different expectations of being academics and mothers to “denote *a unity* of experience, of sex, gender, and desire” (Butler, 2006, p. 30). Notably, it is broadly reported by the interviewed women academics that in the first few years after the infants were born, mothers' time is severely occupied and broken to bits by childcare; thus, their research productivity would drop drastically in these years. In the narrative of Naya, women academics have to take years to recover from this motherhood penalty, but they still have to keep at least an average speed of pace in career development. Therefore, women academics tend to make extra efforts to dispel the hindrances brought by family commitment through spending more personal leisure time doing research (Aiston and Jung, 2015).

As time goes by, as Naya claimed, the effect of motherhood is likely to drop gradually, which means women academics could have increasingly more spare time for their academic careers. However, the interruption of career development may reconstruct their agency and cause the group of Chinese women academics to leak from the academic pipeline. Her explanation may help understand the disproportionate attrition of women in senior academic positions. If a woman academic could survive the “*combo boxing of work, family, and children*” (Naya), she may march forward on her academic path at a faster pace because she regains abundant time. The career path described by Naya reflects the reinforcement of the heterosexual matrix conducted by academic mothers, who are constituted by “an ongoing discursive practice” (Butler, 2006, p. 45), which is undergoing in the zero-sum game between families and careers.

### Ontological responsibility of motherhood

A heterosexual matrix is pervasive in parental duties among Chinese families. In the narratives, many of the women academics agreed that in the first 2 or 3 years, mothers are irreplaceable to children. As they described, “*My daughter only wanted mom when she was little*.” (Ming); “*At that time (from new-born to toddlers), probably some mother's responsibilities cannot be substituted by others*.” (Yang). When Ming and Xixi were asked about their future academic career development, they answered with the take-for-granted attitude that women have to sacrifice their careers for childrearing:

*For the life plan, a good plan is to keep strength in both career and family. In childrearing, the role of women is to be responsible, though it may sacrifice their research productivity*. (Ming)

*My concentration is not on my career development in recent years. Dads cannot do the breastfeeding…I know that people are free to have their choices. However, I just don't have this courage* (Xixi).

The social expectation of being good mothers gives women academics agency to become the major caregivers to children, especially in the first few years after childbirth. However, in these years, none of the interviewed academic mothers claimed their research workload was lighter than that of their colleagues. Therefore, bearing this tension between motherhood and academic career, these academic mothers craved shared responsibility to load the pressure of the “second shift” (Dickson, 2018).

On the contrary, in careless neoliberal institutional culture, the academic working system assumes the employees have no other social responsibility than working, no limitation in working hours, and no interruption in career development (Acker, 1990). The neoliberal academic system is operated with the presumption of degendered academics. When women academics are shaped by gender norms in the Chinese social discourse, it might be difficult for them to fit into this culture. Chinese women academics are expected to take on more family responsibilities, be the main caregivers to families, and accept the disproportional allocation of family affairs in accordance with Chinese traditional gender roles and expectations (Cho and Ryu, 2016; Aiston and Fo, 2021). It contradicts the expectation of ideal neoliberal subjects who are infinitely flexible and always on call and thus will cause the dissatisfaction of employers, helping build the gender hierarchy in Chinese universities. The reproduction of gender is rooted in the culture desire (Butler, 2006). Ming and Xixi took this epistemological construction of gender as ontological and then showed loyalty to maintain “a stable and oppositional heterosexuality” (Butler, 2006, p. 31) by considering the sacrifice of mothers' careers as inevitable. They both prioritized motherhood to their academic careers.

### Ongoing academic career with help

If women academics “*do both [academic career and family] very well, they must have family members to help”* (Yang). In the social discourse, women are regarded as subjects in family life and anyone who cooperate with them are the helps. The dated norms of women associated with childcare are still operating on women academics (Acker and Armenti, 2004). Therefore, to maintain a work–life balance, women academics seek social support from, for example, spouses and parents (Naz and Khan, 2017). Compared with men, women have to rely on exterior help to ensure their devotion to academic career development, for they are still expected to perform their gender roles. Chinese fathers tend to interpret their responsibility of raising children as financial support though they are not the only family providers (Cao and Lin, 2019). This perception of fathers' role as breadwinners mirrors mothers' role of taking the rest childcare responsibility, which is following the narratives of the women academics in this study. The prediscursive allocation of responsibility forms the gender normativity of women.

Han lived with her parents for help with childcare. At the very beginning of the interview, she apologized because her parents happened to go out for a last-minute notice that day. Thus, she had to complete the scheduled interview with an infant in her arms. The interview was hard to precede because of the baby's continuous interruption until her parents got back home. Han was so released, “*Great, they are back*.” This little anecdote reflected the dependence on intergenerational help during childcare to maintain the full-time work pace for academic mothers. The shared caring responsibilities with the grandparents enabled Han to complete the interview.

Some participants reported that they had helps to take care of their children. Kadi took her mother with her to the United States for childcare while she was working in an American university as a visiting scholar. Kadi, her mother, and her child lived in an apartment. When Kadi was taking the interview in the study, her mother was accompanying the child in the living room. Similar to Han, Kadi was able to undertake this interview while her mother was doing the childcare. Han was anxious in the interview before her parents came back, while Kadi was calm and talkative in a light mood. The contrast of their performative acts reflects the reliance on the domestic support of academic mothers, and this cultural inscription on their bodies reduces their autonomy. “*It is my daily research time*,” Kadi told us in the interview when the local time was 11 pm because her mother looked after her child every night to maintain Kadi's academic working time.

With the assistance of family members, Han and Kadi were able to continue with their academic careers under the navigation of gender normativity. It enabled them to perform like careless academics by maintaining the priority of work (Manathunga et al., 2020). As academic mothers, they faced the “either–or” dilemma of being good mothers and successful academics at the same time shaped by the culture that advocates disconnection of work and family (Manathunga et al., 2020). Their professional and personal lives were intertwined compared with male colleagues (Armenti, 2004). Therefore, Han and Kadi had to keep switching their roles between “*competing in academia as men*” (Ming) and being caring mothers as women, “in a form of oppositional heterosexuality” (Butler, 2006, p. 31). This tension that arose out of the hegemonic heterosexual matrix is partially released by shared caring duties, which gives more possibilities for their ongoing academic career.

### Guilt buried in career aspiration

Instead of making more efforts to meet the dual expectations in family and career, Yang was the only academic mother who provided the experiences about how she gradually transferred her childrearing responsibility to her husband and returned to her academic career in the first 3 years after childbirth. Notably, the parental duty of husbands was hardly described in the narratives of the other academic mothers. Similar to the perceptions of Ming and Xixi, Yang was the main caregiver of her daughter after childbirth. She related,

*In the first two or three years [after childbirth], I think I was the one who contributed more [to the family]. The situation overturned after three years…he [my husband] felt good during the process [of childcare]. He enjoyed the happiness of taking an important role and being a caregiver, which gave him a sense of existence and value…Because I am the kind of person who values career development very much, I give it a priority. If I am asked to make a choice [between family and career], I will definitely prioritize career development*. (Yang)

Yang emphasized, “*the identity of women has a negative impact on academic career development*.” She identified herself as a career woman and devoted an enormous amount of time to her research project during the pandemic, while her husband, who is also an academic, took care of their 6-year-old daughter and family affairs. The reconstruction of motherhood reduced the pressure on Yang and then empowered her husband in childrearing, which was indeed the empowerment of herself in career development, saving her more time to do research. It shows when Yang spent more time on her academic career, her husband needed to take on more childcare responsibility to maintain the heterosexual matrix.

Academic mothers' research productivity drops drastically during the COVID-19 pandemic (Vincent-Lamarre et al., 2020) mainly because they dedicate more time to domestic activities and childcare than their partners do (Lagomarsino et al., 2020). However, Yang's research productivity raised like men. In 2020, Yang and her research team made tremendous research achievements by having publications in the highest ranking international journals. Because of her social contribution to her professional field, she received the provisional award of “Anti-pandemic pioneer.” Despite her achievement, and although she took fewer childcare responsibilities than the first few years after childbirth, she still desired to meet social expectations. She frequently posted photos and stories of her daughter as well as her academic work on a social media platform to perform the normativity of being a perfect mother and accomplished academic simultaneously. She expressed complex feelings,

*I am willing to do both (childcare and academic work) well. Sometimes if I did not do this (childcare) well enough, I would feel quite guilty and blame myself. Thus, I have been living in a very contradictory situation…I think it is similar to [academic] work. Since I am responsible for the job (motherhood), I have to do this well. (Yang)*

The situation that Yang was in this “*contradictory situation*” mainly showed how “the binary regulation of sexuality suppresses the subversive multiplicity of a sexuality” (Butler, 2006, p. 26). Yang clarified to us that she wanted to be a good mother but emphasized her academic career development as well. She then displayed the presence of motherhood to reproduce this “figure of fantasy” (Butler, 2006, p. 184) to be a good mother under the discipline of social discourse. Notably, Yang expressed guilt, worry, and anxiety on the concern of not being a good mother when she showed the subversion of a multiplicity of sexuality (Butler, 2006) by transferring the caring duty to her husband. By contrast, Han and Kadi did not show similar negative feelings at all when they received help in childcare. A tentative explanation might be Han and Kadi gained assistance from their mothers, who are also females, which kept their performances still within the frame of the heterosexual matrix. However, when Yang was not the main caregiver to her daughter anymore, the labor division between her and her husband showed inconsistency with the normative heterosexual matrix. The displacement of Yang's “female duty” made her feel distorted so that she was constantly stuck with contradictory circumstances.

## Discussion: Performances of academic mothers

Neoliberalized universities emphasize stressful work and academic excellence (Rosa and Clavero, 2020). Rooted in this culture, hegemonic masculine ideologies are perpetuated and reproduced in highly competitive academia, in which women academics are more likely to experience the work–life conflict and its associated impacts (Blithe and Elliott, 2020; Rosa, 2022). In this study, most Chinese academic mothers tended to fulfill their family responsibilities first to satisfy the expectation formed in the heterosexual matrix. When these women academics were shaped by the two distinctive sets of contested normativity at the same time, they had to properly reposition themselves. As follows, we argue that to respond to the gendered exclusion formed in male-dominated social and institutional discourses, Chinese academic mothers tend to conform to gender normativity, but they also seek their career advancement under this tension through the fluidity of gender.

First, many Chinese academic mothers reported gender exclusion in male-dominated social and institutional discourses. It is the heterosexual matrix that builds this exclusion when women academics are expected to perform gender normativity. As a structural problem, women are, to some extent, unwelcome in masculine academia (Banchefsky and Park, 2018). According to the interview data, if these academic mothers were willing to stand out in this masculine competition, they must exclude their femininity to fit into the careless institutional culture of neoliberalism. That is to say, academic mothers need refuse their “female other” (Butler, 2006, p. 72) and identify themselves as men to survive the academic competition. Another layer of explanation might be the ongoing tension Chinese academic mothers experience, which originated from the heterosexual matrix, between family and career. In the contested discourses, they need to perform differently to meet the dual normativity. They attempt to make boundaries in the heterosexual matrix permeable to be academically successful in male-dominated institutions when they perform gender domestically to stay in sites they *ought to be*. As Butler (2006, p. 184) explains, “the construction of the gendered body through a series of exclusions and denials, signifying absences.” For Butler, when the academic mothers perform femininity in the family, they are excluding masculinity, and vice versa. The “exclusions and denials” (Butler, 2006, p. 184) make the family and career of academic mothers a zero-sum game, as explained in the findings. In this game, academic mothers need to demonstrate femininity and masculinity interchangeably to fit in the two sets of normativity. Although what academic mothers show in either family or career is in “temporal and collective dimensions” (Butler, 2006, p. 191), they are still constrained in the heterosexual matrix with gender exclusions.

Second, the performances of these Chinese academic mothers were formed by gender normativity in the heterosexual matrix. Many interviewees pictured their ideal lives as “*a stable job, and a happy family*” (e.g., Shi). For this reflection of normativity, these women academics developed performative acts to balance the expectation. When women academics started careers, they kept wandering back and forth between families and workplaces to perform as what they were expected to own “*a happy family*” and thus gave up some opportunities in career development. For example, Rui said she could hardly attend academic conferences when her daughter was little. On the other hand, albeit these women academics performed gender in their academic paths (e.g., Macfarlane and Burg, 2019), they are willing to equip themselves by performing “*like men*” (Ming) to compete for the opportunity of “*stable jobs*” in career development. For example, Yang took fewer responsibilities in childcare after her child became older. For Butler (2006, p. 191), the performance of normativity is not individual but collective, and it helps maintain gender within the binary frame. The gendered collective performative acts in male-dominated social and institutional discourses among academic mothers enhance the heterosexual matrix. In the constant zero-sum negotiation, academic mothers constantly struggle with the work–life conflict, which sometimes results in their hesitation and confusion in career advancement.

Third, gender fluidity was shown in their wrestling between family and career. In the resistance and subversion of gender normativity, some of these Chinese academic mothers reallocated responsibilities in the frame of heterosexuality. For example, Han and Kadi sought help from their parents, and Yang transferred the primary childcare responsibility to her husband. For Butler (2006, p. 188), “[t]his perpetual displacement constitutes a fluidity of identities that suggests an openness to resignification and recontextualization.” Although this fluidity of caring duties temporarily set academic mothers free from gendered domestic constraints, it does not reconstruct the heterosexual matrix. Instead, it shows its solidarity. These Chinese academic mothers may have possibilities to reposition themselves to fit in the careless institutional culture, but it indeed enhanced the gender normativity in both family and career by giving *femininity* to other family members based on the heterosexual matrix. Han and Kadi's parents and Yang's husband took over their feminine, childcaring roles. For Butler (2006, p. 188), the childcaring duties are considered “a set of imitative practice.” These Chinese academic mothers did not reconstruct but enhanced the heterosexual matrix because the gendered labor division was still clear and stable. However, they might still be seen as the ones who challenged the gender normativity when they reloaded their gendered responsibilities and *intruded* in academia which has been operated in masculine ethics for decades (Sheppard, 1989; Zippel, 2017), limiting women's access and opportunities (Banchefsky and Park, 2018). Therefore, this group of academic mothers inexplicably “returns the glance, reverses the gaze, and contests the place and authority of the masculine position” (Butler, 2006, xxx) by presenting a subversive pose. These possibilities of fluidity in the heterosexual matrix unravel the fictiveness of gender and thus lessen the “naturalized or essentialist gender identities” (Butler, 2006, p. 188).

## Conclusion

This study seeks to explain how Chinese academic mothers perform in the hegemonic heterosexual matrix constructed by male-dominated social and institutional discourses. Based on the qualitative analysis of the narratives from six Chinese women academics, we conclude that they were involved in a zero-sum game of motherhood and career advancement. From the findings, we identify the critical issue of this study: in the masculine academia, these women academics helped maintain the heterosexual matrix by satisfying the gender normativity when they negotiated their performances in family and career; meanwhile, most have developed some strategies to achieve their career advancement.

This qualitative study has several application implications that may contribute to the field. For one, for the Chinese educational administrators and policymakers, we call for more measures to help reduce the motherhood penalty and to support the career advancement of Chinese academic mothers. For another, this study shows what challenges Chinese academic mothers are likely to face in their career progression and provides examples and analysis for early-stage women academics and particularly academic mothers who may be enlightened when they are in the dilemma.

With these implications, we also recognize the limitations of this study. This small-scale qualitative study may not be generalized to show the whole picture of Chinese academic mothers. Another limitation lies in the lack of psychological intimacy *via* online interviews (Denscombe, 2014) we conducted for the reason of the pandemic because remoteness may restrict the participants' expression to some extent. Future research may quantitatively explore the work–life balance of Chinese academic mothers by including more participants from different levels of universities, and their strategies to cope with it.

## Data availability statement

The raw data supporting the conclusions of this article will be made available by the authors, without undue reservation.

## Ethics statement

The studies involving human participants were reviewed and approved by Granting body: University of Auckland Human Participants Ethics Committee Reference Protocol Number: 024731. The patients/participants provided their written informed consent to participate in this study.

## Author contributions

LB: conceptualization, methodology, formal analysis, investigation, writing—original draft, writing—review and editing, and project administration. GW: validation, resources, and writing—review and editing. All authors contributed to the article and approved the submitted version.

## Funding

The research leading to these results has received funding from the Ministry of education of Humanities and Social Science project (Grant Number 22YJC840001), the Project of Social Science Foundation of Jiangsu Province (Grant Number 22JYB024), and the Educational Science 14th Five Year Plan Research Program of Nanjing University of Posts and Telecommunications (Grant Number GJS-XKT2108).

## Conflict of interest

The authors declare that the research was conducted in the absence of any commercial or financial relationships that could be construed as a potential conflict of interest.

## Publisher's note

All claims expressed in this article are solely those of the authors and do not necessarily represent those of their affiliated organizations, or those of the publisher, the editors and the reviewers. Any product that may be evaluated in this article, or claim that may be made by its manufacturer, is not guaranteed or endorsed by the publisher.
